# Economic Impact of Managing Acute Diabetic Foot Infection in a Tertiary Hospital in Malaysia

**DOI:** 10.5704/MOJ.1403.018

**Published:** 2014-03

**Authors:** AWC Lam, MR Zaim, HH Helmy,, IMA Ramdhan

**Affiliations:** Department of Orthopaedics, Hospital Sultanah Nur Zahirah, Kuala Terengganu, Malaysia; Department of Orthopaedics, Hospital Sultanah Nur Zahirah, Kuala Terengganu, Malaysia; Department of Orthopaedics, Hospital Sultanah Nur Zahirah, Kuala Terengganu, Malaysia; Department of Orthopaedics, Hospital Sultanah Nur Zahirah, Kuala Terengganu, Malaysia

## Abstract

Diabetic foot disease is the leading cause of non-traumatic amputations of the lower limb, hence a major health care and socioeconomic burden. It has been found that most of the costs occur in the inpatient setting; therefore this study is to quantify the costs of managing inpatient diabetic foot infections (DFI). We treated 182 inpatients from May 2012 till April 2013 and analysed the cost of antibiotic usage, wound dressing, surgical procedure, admission and basic investigation costs. The total cost was ~ USD 11,000 (2013). This number, however, only reflects the cost for managing an acute infection. The price for follow-up care as an outpatient, rehabilitation and indirect costs (emotional suffering, reduced productivity) is estimated to be much more.

**Key Words:**

Economic impact, diabetic foot infection

## Introduction

Diabetes and its complications are quickly becoming an epidemic in Malaysia. As globalization is inevitable, we inherit the risks of non-communicable diseases, which have overwhelmed developed nations. This poses as a major threat to the development of this country as the demographics of this disease is shifting towards the youth. It is estimated that 2.6 million Malaysians are diabetics, 15.2% of our total population, and these numbers have been projected to increase sharply over the years^1^.

Diabetic foot disease is said to affect 15 to 25% of diabetics in the course of their lives^2-4^ and is the leading cause of nontraumatic amputations of the lower limb^5, 6^. This significant diabetic complication not only affects the patient, but their families and our country, and will eventually lead to exorbitant treatment costs, hampering the growth of our country.

Reiber et al found that most of the costs occur in the inpatient setting7. However, these costs were based on international demographics. Up to date there is no recent analysis to objectively estimate the cost in managing inpatient diabetic foot infection. We therefore conducted this study to quantify the financial impact of managing patients admitted for diabetic foot infection (DFI) in a tertiary hospital in Malaysia, specifically the cost of antibiotic usage, surgical procedure and wound dressing.

## Materials and Methods

### 

**Patients**We retrospectively analysed 182 diabetic patients who have been admitted to Hospital Sultanah Nur Zahirah (HSNZ), Terengganu, Malaysia, for DFI from May 2012 till April 2013. Data was collected from our Diabetic Inpatient Registry and included patients who have been admitted due to acute DFI and was treated by either conservative methods (wound dressing and antibiotics) or combined with surgical intervention (debridement or amputation). Exclusion criteria were diabetic patients with foot infection who were admitted to wards of other medical disciplines (Intensive Care Unit and medical wards) due to other concurrent illnesses, patients who have been managed as an outpatient, and those who have an incomplete diabetic registry.

**Costs Analysis** *Antibiotic Usage*We referred to the National Antibiotic Guideline 20088 for selection of antibiotics for treatment of DFI. After the culture and sensitivity was made available, the antibiotic was changed accordingly. The common antibiotics used for the 12-month period were analysed and its costs were calculated based on total vials or doses given per day to the patients either intravenously (IV) or per oral (PO): IV Sulbactam – ampicillin (Unasyn) 1.5g TDS, IV Cefuroxime 750mg TDS, IV Ceftazidime 1g BD, IV Metronidazole 500mg TDS, IV Cloxacillin1g QID, PO Fusidic acid 500mg TDS, IV Vancomycin 500mg TDS, IV Ciprofloxacin 400mg TDS and IV Gentamicin 80mg BD. Patients who were admitted were started on recommended antibiotics and subsequently changed according to tissue culture and sensitivity. We referred to our inpatient pharmacy for the cost of antibiotics^9^. (Table I)

*Surgical Procedure*Patients who presented with moderate to severe DFI required surgical intervention to eradicate the source of infection. Provided consent was obtained, surgical procedures were either local tissue debridement or amputation (major: Transfemoral and Transtibial, minor: Ray amputation and wound debridement). The cost for each surgical procedure was standardized and documented in National Fee (Medical) (Amendment) 2003^10^. The cost of each surgical procedure differed depending on the admission of a patient to different ward classes (first, second and third); for the purpose of this study, patients were collectively charged as third class. (Table II)

*Wound Dressing*All DFIs require wound dressing. The selection of type of dressing solution used for each wound was based on wound condition either at its presentation on admission or after surgical procedure. We analysed common types of dressing solutions and materials used over the 12-month period. For the purpose our study, the average wound size taken was 5x10cm, and was dressed once a day. Each conventional dressing was done using 30mL of solution, using a pack of gauze (10 pieces in each) and a disposable tray. For advanced dressings (eg: Intrasite gel), usage were as recommended by the manufacturer. The cost for each dressing solution and material was obtained from our pharmacy9.(Table III)

*Others*The admission cost is defined as: cost of hospital stay (chargeable per day), hematological and foot radiological investigations, which were done for all patients who had been admitted with DFI. For the purpose of this study, all patients are charged as admission to third class wards. Our calculations involved per day admission charges and a one off initial charge for hematological investigations (Full10 Blood Count (FBC), and Blood Urea Serum Electrolyte/Creatinine (BUSEC)) and foot radiographs (2 views; anteroposterior and lateral views)^10^. (Table IV)

## Results

A total of 182 patients who were admitted for management of DFI over a twelve-month period were identified from our hospital diabetic registry. One hundred and eighteen (64.84%) of our patients were male, while sixty-four (35.16%) were female. Malay patients predominated in our study, comprising of one hundred and eighty patients (98.90%). The other two patients (1.10%) were Chinese. We also found that majority of our patients, one hundred and eleven (60.99%), had diabetes for 5 years or more, whereas seventy-one (39.01%) of them had less than 5 years of diabetes. At most, we had 26 patients admitted per month for treatment of DFIs, with a mean of 15 patients per month. The total admission period for 182 patients was 1, 781 days, giving an average of 9.79 days per patient.

The antibiotics and their doses were calculated on the basis of recommended treatment8. Being established as the empirical antibiotic of choice for DFI8, sulbactam-ampicillin was most commonly used. The total cost for antibiotic used over a period of 12 months was ~ USD 5,400 (2013).

From our study sample, one hundred and thirty-one patients (71.98%) needed surgical intervention. From that group, fifty-four (41.22%) patients underwent an amputation. Amongst the amputees, sixteen patients (29.63%) had a major amputation, whereas thirty-eight (70.37%) had a minor amputation (Ray amputation). The total cost for surgical intervention over the period of 12 months was ~ USD 1,000 (2013).

With regards to dressings, Dermasyn and Normal Saline dressing proved to be the two most frequently used. The total cost for dressings, including the required disposable instrument set was ~ USD 1,900 (2013).

Admissions and baseline investigation costs for our study sample was ~ USD 2,400 (2013).

The total cost for managing 182 inpatients with DFI over 12 months in HSNZ was ~ USD 11,000 (2013) (Table V). From this amount, half of the expenditure (50.29%) was attributed to antibiotic use, significantly more than the nine percent for surgical procedures. Admissions and baseline investigations, together with the cost for wound dressing contributed to the rest (40.68%) of the total expenditure, at 22.73% and 17.95% respectively.

## Discussion

In general, the costs involved in managing diabetic foot infection includes acute management of the infection as an in-patient, continuing treatment as an out-patient in the primary health clinics and other miscellaneous costs at home. Our data highlights the costs of managing acute diabetic foot infection in the in-patient setting. This translates to ~ USD 11,000 per year and USD 60 (n=182) per patient per year (adjusted to USD 1= RM3.23, based on Bank Negara Malaysia on Ringgit Foreign Exchange Rate; dated November 2013). In comparison to other literature, Ragnarson Tennvall et al estimated in 2004 that it costs USD 17,500 to heal a single ulcer, and up to USD 33,500 when lower extremity amputation is involved^11^. Their cost not only includes inpatient hospital care, surgery, investigations and antibiotics, but also, regular visits to the podiatrist, orthopaedic appliances and outpatient topical treatment until resolution of the ulcer which may take months or years. The costs of managing any disability or recurrence of the initial ulcer were also included.

While the above number may not seem alarming, it is noteworthy that this is only the cost of acute DFI treatment, in one of the 14 main tertiary hospitals in Malaysia. With the design of our economic and health care system, our hospitals benefit from heavy subsidization from the government, making the true cost of healing an ulcer even more substantial. Our government allocated ~ USD 6.8 billion in 2014 for healthcare alone ^14^, although this amount is distributed through multiple channels, the burden of diabetic foot infection is still significant.

We also found that the majority of our cost arises from the administration of antibiotics. As most of the organisms isolated from a DFI is polymicrobial^7^, it is wise to use a broad spectrum antibiotic, like sulbactam-ampicillin, as empirical therapy. Sulbactam-ampicillin is also the cheapest antibiotic in our group. This led to it contributing the most to the antibiotic group cost, not surprising, as it is the most commonly used antibiotic. Antibiotics are also the first line treatment for active DFI^8^. With oral antibiotics being sufficient for mild infections, moderate to severe infections may require multiple groups of parenteral antibiotics for a longer duration^7, 8^, whilst supplemented with surgical debridement. Ultimately, this makes the antibiotic group the largest contributor to the cost of DFI management.

Surgical procedures contributed the smallest percentage to our cost. Religious and community factors are key to the above result; in Terengganu many patients view amputation as a taboo. The social stigma that losing a limb makes one “paralysed” leads them to choose solely antibiotic treatment over surgical intervention despite medical advice^12^. As the vast majority of our patients are Muslim, some believe to be buried whole^13^, hence refusing life-saving amputations. This delicate situation requires doctors and associated healthcare professionals to take the time to correct these fallacies, aiding a patient in choosing his life over his limbs. Forming peer support groups to include fellow amputees can also boost a patients’ morale, giving them an insight to the eventual functional outcome one can achieve.

Wound dressing plays a significant role in the treatment of DFI and cost us approximately 18% of our total expenditure. Similar factors as stated above contribute to the cost of wound dressings: patients in our review preferring the more conservative approach in treatment. Dermasyn was the most popular choice for wound dressing. That result is expected, as it is the cheapest alternative for chemical debridement, making it readily available in the hospital.

Admission and basic investigations cost ~ USD 2,400 (2013) for 182 patients. These are the expenses incurred before initiation of any form of treatment. This figure did not take into account other investigations (blood or swab for cultures and analysis, chest radiographs, electrocardiograms, for some an echocardiogram, as preoperative assessment), and, for simplification, only included a one off charge for these investigations. Thus, contrary to the reality that some of these patients with multiple comorbidities would probably have more investigations done. The other major hidden costs which were not included in the study involve admission to the intensive care unit and medication costs like insulin therapy.

Our study only takes into account the direct costs of DFI, while the indirect costs were more subjective and difficult to quantify7. This includes the costs of outpatient treatment,transportation, dressing and psychological impact to the patients that in truth are more burdening to the patient(4). Further studies are needed to evaluate the true cost of DFI including prospective studies to monitor a cohort of patients from presentation, beyond discharge from the hospital, till the resolution of their ulcer.

**Table I: Antibiotic cost:**
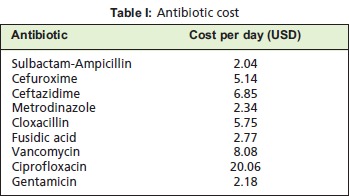


**Table II: Surgical procedure cost:**
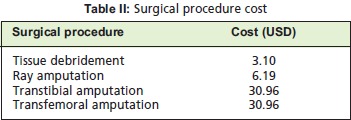


**Table III: Dressing cost:**
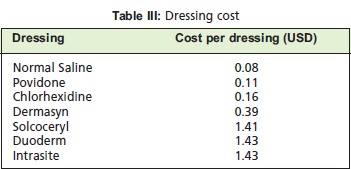


**Table IV: Admission and baseline investigation cost:**
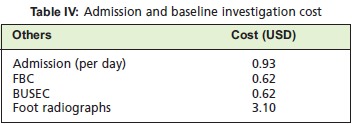


**Table V: Total cost over 12 months:**
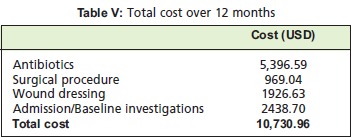


## Conclusion

In order to treat DFI, multiple modalities of treatment should be used concurrently: antibiotics, adequate surgical debridement as well as good dressing technique. Executing these three components well, the costs can be substantially reduced, thus lessening the economic impact to the government, and more importantly the patients themselves. Access to podiatrists and regular follow-up post discharge can also ensure compliance to medication and dressing techniques, thus reducing the recurrence of complications. 

Also, much can be done to raise awareness at the root level. We found that many patients, despite having diabetes for 5 years or more, do not have insight to their own illness, let alone its complications. Substantial amount of money has been spent for management of DFI, which in essence, with proper care, is preventable. Resources should be allocated towards awareness campaigns at not just a national level, but the local community setting. Prevention is better than cure.
